# Identification of cassava germplasms resistant to two-spotted spider mite in China: From greenhouse large-scale screening to field validation

**DOI:** 10.3389/fpls.2022.1054909

**Published:** 2022-12-07

**Authors:** Xiao Liang, Qing Chen, Ying Liu, Chunling Wu, Kaimian Li, Mufeng Wu, Xiaowen Yao, Yang Qiao, Yao Zhang, Yue Geng

**Affiliations:** ^1^ Environment and Plant Protection Institute, Chinese Academy of Tropical Agricultural Sciences, Key Laboratory of Integrated Pest Management on Tropical Crops, Ministry of Agriculture and Rural Affairs, Haikou, Hainan, China; ^2^ Sanya Research Academy, Chinese Academy of Tropical Agriculture Science, Hainan Key Laboratory for Biosafety Monitoring and Molecular Breeding in Off-Season Reproduction Regions, Sanya, Hainan, China; ^3^ Tropical Crops Genetic Resources Institute, Chinese Academy of Tropical Agriculture Sciences, Haikou, China

**Keywords:** *Manihot esculenta*, *Tetranychus urticae*, resistance, mite pest, eco-friendly

## Abstract

**Introduction:**

Utilization of resistant germplasm is considered as an effective, economical and eco-friendly strategy for cassava pest management. *Tetranychus urticae*, known as the two-spotted spider mite (TSSM), is a devastating pest in Asian cassava planting countries as well as in China. However, the resistant levels of abundant cassava germplasms to TSSM remains largely unknown.

**Methods:**

To fill this knowledge gap, we conducted screening of 202 cassava germplasm for resistance to TSSM in China based on the classification of mite damage phenotype, under both greenhouse and field conditions.

**Results:**

The three rounds of large-scale greenhouse experiments had identified two highly resistant (HR) varieties (C1115 and MIANDIAN), five resistant (R) varieties (SC5, SC9, SC15, COLUMBIA-4D and LIMIN) and five highly susceptible (HS) varieties (KU50, BREAD, SC205, TMS60444 and BRA900), besides, these ‘HR’ and ‘R’ varieties would significantly repress the normal development and reproduction of TSSM. In addition, the 12 cassava varieties selected from the greenhouse screening were further subjected to consecutive five years of field validation at Danzhou, Wuming and Baoshan. The seven resistant varieties not only exhibited stable TSSM-resistance performance across the three field environments, but also possessed the same resistant levels as the greenhouse identification, while the resistant varieties SC5 was an exception, which was identified as moderate resistant in Baoshan, indicating the variety-environment interaction may affect its resistance. Furthermore, regional yield estimation suggested that the higher the resistance level was, the better capacity in reducing the yield losses.

**Discussion:**

This study demonstrated that the TSSM-resistant varieties could be considered as ideal materials in mite control or in future breeding programme of mite-resistant cassava plant.

## Introduction

Cassava (*Manihot esculenta* Crantz), serving as food, animal feed as well as biomass energy (Wu et al., 2022), is widely cultivated in more than 100 countries (Parmar et al., 2017). In China, this crop is mainly used for the production of ethanol fuel, which accounts for approximately 70% of the consumption (Jiang et al., 2019). In the past couple of years, China is the world’s largest importer of cassava, while most cassava products such as chips and flour were imported from Southeast Asian countries, *i*.*e*., Thailand, Vietnam, Laos, and Cambodia (Tan et al., 2018). However, only a few provinces located in the south and southwest of China possess suitable growing conditions for cassava cultivation. Therefore, increasing the yield will be an important demand for increased ethanol production (Chen et al., 2016). Moreover, in order to ensure sufficient profit, farmers prefer a robust cultivar and easy-handling field management strategy.

Insect pest is of great threat to cassava yield, and the phytophagous mite is one of the most destructive pests (Chen et al., 2022a). The cassava green mite (CGM), *Mononychellus* spp., is one of the most widely distributed cassava pests in the world (Vásquez-Ordóñez and Parsa, 2014). Comparatively, the *Tetranychus* spp., known as red spider mite, were predominantly distributed in Asian countries. There were over 10 species found in cassava fields (Bellotti, 2008). In particular, the two-spotted spider mite (TSSM; *Tetranychus urticae;* Acari: Tetranychidae) can cause 50%–70% yield losses in China (Chen et al., 2019). To date, acaricide application is still the major strategy to control TSSM. However, the dense canopy of the cassava plant makes it difficult to target the acaricide. Moreover, the excessive use of acaricide may also largely reduce the natural enemies’ population and bring about mite resistance problems. Up to now, the cases of TSSM resistance to several acaricides are continuously increasing, including acequinocyl (Leeuwen et al., 2008), spirodiclofen (Van Pottelberge et al., 2009), and cyenopyrafen (Khalighi et al., 2016).

Utilization of resistant germplasm is considered as an effective, economical, and eco-friendly strategy for cassava mite management. Compared to breeding a novel mite-resistant cassava plant, identification of the resistance level of existing cassava germplasms is much more convenient and efficient. Several organizations like the International Center for Tropical Agriculture (CIAT) and the International Institute for Tropical Agriculture (IITA) have made tremendous labors to evaluate cassava resistance to insect pests like CGM, whitefly, and thrip (Bellotti et al., 2012)—for example, MEcu 72, a cassava genotype from CIAT, has been identified as resistant to *Aleurotrachelus socialis* (Bellotti and Arias, 2001; Carabalí et al., 2010a; Carabalí et al., 2010b), *Bemisia tuberculata* (Barilli et al., 2019), and *B. tabaci* (Omongo et al., 2012). In addition, some genotypes from South America and Africa posed different levels of resistance to *B. tabaci* (Omongo et al., 2012; Ochwo-Ssemakula et al., 2019). In a similar study at the IITA in Nigeria, two cassava genotypes supported the lowest number of whiteflies (Ariyo et al., 2005). By comparison, studies focusing on identifying mite-resistant materials in cassava populations are relatively limited, and most studies were focused on screening resistant materials against cassava green mite (CGM). Over 300 accessions from the CIAT Columbia germplasm were shown to have some degree of resistance to CGM (Bellotti et al., 1999). At IITA Tanzania, 58 clones were observed to have a distinct resistance level to CGM (Bellotti et al., 2012). In addition, evaluations of cassava resistance to different insect pests were also conducted. Parsa et al. (2015) performed 89 field evaluations of cassava landraces resistant to several insect pests and found that 129 landraces were highly resistant to thrips, while 33 landraces were resistant to CGM, and 19 landraces were resistant to whiteflies.

CGM sporadically emerged in China (Lu et al., 2014), while the TSSM is the predominant cassava mite; therefore, it is more imperative and practical to develop a TSSM-resistant variety. However, field identifications of cassava resistant to *Tetranychus* mite were rather limited compared with those focused on CGM, which was hindered by the low population in certain cassava-planting regions, for example, the CIAT in Colombia (Bellotti et al., 2012). Nevertheless, laboratory identifications still have been carried out, and mite mortality and hatching rate were used as key indexes for evaluating the resistance of cassava plants—for instance, while fed on the resistant cultivars MBra 12 and MCol 1434, the mortality of TSSM larvae and nymphs was 68% and 50% higher than those on the TSSM-susceptible cultivar MCol 22, respectively. Moreover, the hatching rate and the survival rate of larvae were significantly lower on resistant cultivar MCol 1351 than on MCol 22 (Bellotti et al., 2012). On the contrary, identification of cassava germplasm resistant to *Tetranychus* species is recommended to be conducted in regions with high mite populations. Asian countries that cultivated cassava and suffered huge economic losses provide a good research opportunity (Bellotti et al., 2012). Based on leaf morphology, secondary metabolites, and proteomic analysis, Yang et al. (2020) found that the cultivar Xinxuan 048 exhibited high resistance to *T. cinnabarinus* under both greenhouse and field conditions.

However, as far as we know, there is a lack of study regarding either the laboratory or field screening of TSSM-resistant cassava varieties from large populations. To fill this knowledge gap, in the present study, 202 cassava germplasms including all the main cultivars in China were subjected to three rounds of large-scale greenhouse screening. Furthermore, the identified resistant and susceptible varieties from the preliminary screening were validated for their field performance at three different regional sites in five consecutive planting seasons. We expect to screen cassava germplasms with stable mite-resistant performance and provide promising materials for either mite control or future breeding programs of mite resistance.

## Materials and methods

### Cassava germplasms

A total of 202 cassava germplasms were derived from the National Cassava Germplasm Nursery of China, Chinese Academy of Tropical Agricultural Sciences (CATAS). Cassava stems of about 20 cm in length were vertically planted with nutritive soil (equal quantity of soil, peat, and perlite) in pots and grown in a greenhouse for TSSM resistance screening. The light/dark photoperiod was set as 14/10 h, the temperature was maintained at 28 ± 1°C, and the relative humidity was kept at 75 ± 5%.

### Laboratory rearing of TSSM

TSSM rearing was conducted based on our previous study (Liang et al., 2017). Healthy adults were maintained by the Environment and Plant Protection Institute, CATAS, and reared on the back of healthy cassava leaves of BRA900 cultivars at 28 ± 1°C, 75 ± 5% relative humidity, and L14:D10 photoperiod. A water-saturated blotting paper strip was wrapped around the leaf margin to prevent the escape of mites and to keep the leaves fresh. The leaves were replaced every 2 to 3 days.

### Identification method of cassava resistance to TSSM

**Figure 1:**
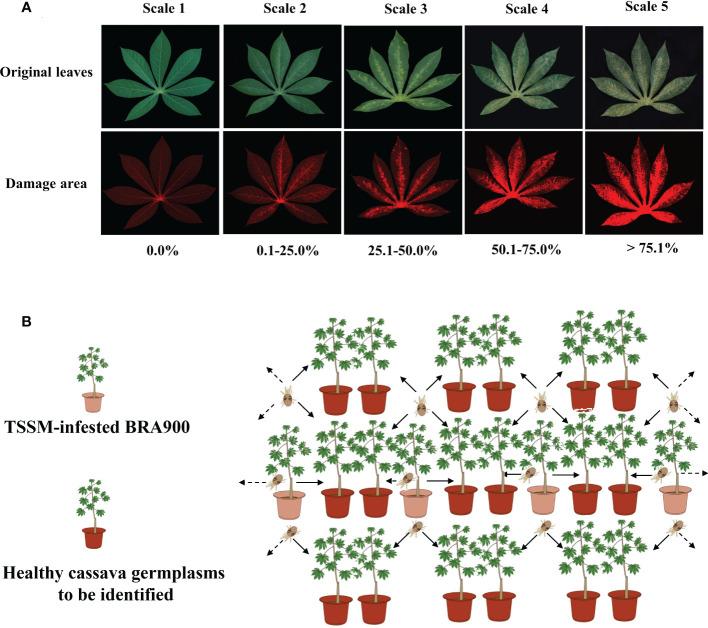
Methodology of identification of cassava germplasm resistance to two-spotted spider mite in greenhouse. **(A)** Classification of mite damage scales of cassava leaves. The upper panel indicated the original leaf images, and the lower panel indicated the mite damage area that was visualized by specialized leaf image analysis software. **(B)** Sketch map of mite inoculation in greenhouse identification.

Identification of cassava resistance to TSSM was based on the leaf damage symptoms caused by TSSM. The mite damage symptoms were classified into five scales (**Table 1** and **Figure 1A**) and first evaluated based on the leaf damage rates. The leaf damage rate was precisely calculated using Leaf Image Analyser (YMJ-E, Daji Co., Ltd., Hangzhou, China) (**Supplementary Figure S1**). After that, the mite damage index (MDI) was calculated according to the equation shown below:


$$\text{MDI}=\frac{\sum \left(S\times N_{s}\right)}{N\times 5}\times 100$$

**Table 1:** Leaf damage scale classification and definition.

Leaf damage scale	Definition
D_0_	No leaf damage
D_1_	Minor leaf damage with sporadic white spots; the damaged area accounts for 0.1%–25.0% of the whole leaf
D_2_	Minor coherent mite feeding marks; the leaf’s damaged area accounts for 25.1%–50.0% of the whole leaf
D_3_	The damaged area covers most of the leaf, the leaf appearance seems chlorisis, and the damage area accounts for 50.1%–75.0% of the whole leaf
D_4_	The damaged area covers the entire leaf, the leaf demonstrates severe chlorosis symptoms, and the damaged area was beyond 75.1% of the whole leaf.

where *S* indicates mite damage scale, *N*_s_ indicates the number of damaged leaves at a certain damage scale, and *N* indicates the total number of investigated leaves. Finally, the six mite resistance levels were identified based on the MDI ranges (%), which were listed as HR (with MDI ranging from 0.1% to 12.5%), R (with MDI ranging from 12.6% to 37.5%), moderately resistant (MR, with MDI ranging from 37.6% to 62.5%), susceptible (S, with MDI ranging from 62.6% to 87.5%), and highly susceptible (with MDI beyond 87.5%).

### Greenhouse study to identify the cassava germplasms resistant to TSSM

Greenhouse identification was conducted at the Key Laboratory of Integrated Pest Management on Tropical Crops, CATAS. A total of 202 cassava germplasms, with each germplasm consisting of three replicates and each replicate consisting of six plants, were simultaneously used for each experimental setting, and a completely random design was used in glasshouse screening. All cassava germplasms were planted in pots in a greenhouse as previously described. After 3 months, the cassava germplasms were used to evaluate resistance to TSSM. For artificial TSSM infestation, a single cassava variety BRA900 which had previously been infested with identical adult TSSM per plant (approximately 500–600 mites) was placed between the cassava test pots, and it was made sure that the infested plants attached to the healthy ones so that the mites were allowed to move naturally between plants. In this setup, six cassava plants were exposed to two infested plants (**Figure 1B**). After 8 days post-mite infestation (dpi), the three most seriously infested mature leaves (judged from the phenotypical symptoms) were sampled and evaluated in terms of TSSM resistance level as per the method mentioned above; therefore, there were 18 leaves for each replicate of each germplasm. The greenhouse identification was conducted for three consecutive rounds (in the year 2015), and each round of experiment took about 4 months.

### The effect of identified mite-resistant and mite-susceptible cassava germplasms on the reproduction and development of TSSM

The cassava germplasms which were identified to be resistant and susceptible to TSSM were selected to evaluate their performance on the reproduction and development of TSSM. Fifty female adults (1 day old) were placed on the leaf back of each cassava germplasm. The mortality was recorded every day until 8 dpi. In addition, eggs laid within 24 h remained on the leaf. For TSSM development observation, the individual newly emerged larva was placed on the surface of the cassava leaf grids, which was divided by using water-saturated blotting paper strip. The developmental duration of eggs, protonymph, deutonymph, and female adults of F_0_ TSSM was investigated every 12 h, and at least 50 tested mites were observed per cassava germplasm. Moreover, the fecundity and the egg hatchability of a single female adult were observed on a leaf from the cassava germplasm until the adult died. The average egg number of each female together with the hatching rate of F_1_ TSSM were recorded using a microscope. Fifty individuals divided into three groups were observed for each cassava germplasm.

### Field identification of cassava variety resistant to TSSM

Experiments were performed in the field located at Danzhou, Wuming, and Baoshan, respectively (the geographic information and soil properties of these three regions are listed in **Supplementary Table S1**). These three experiment sites were the perennial epidemic area of TSSM; hence, the TSSM population was allowed to spontaneously accumulate in the field test without artificial inoculation. Weather elements, *i*.*e*., rainfall, relative humidity, and temperature, were analyzed according to the data recorded by the weather stations located in the experiment sites (**Supplementary Figures S2–S7**).

Before formal field identification, a mite population survey was first conducted on identified “HS” cassava variety BRA900 at the three experiment sites (in the year 2016); the purpose is to confirm the precise inspection time for identification of cassava resistance to TSSM. The survey was carried out 1 month after planting until harvest and on the 5th and 20th of each month (twice a month). The months of mite population peak at those three regions (about 2 months after planting) were recorded and considered as the propriate time for further mite resistance identification (six times for each planting season). In addition, the survey was continuously carried on with the mite resistance identification to ensure the reliability of the identification results in each tested year.

The consecutive 5 years of field identifications were carried out from 2017 to 2021, and 10 months was required from planting to harvest (the schedule can be seen in **Supplementary Table S2**). The treatment plots for each variety were 8 m × 2 m (16 m^2^) in a randomized complete block design (**Supplementary Figure S8A**). Cassava stem segments were planted at a distance of 1.0 m between rows and 0.8 m between plants in the row, and each plot consisted of three rows (30 cassava plants) (**Supplementary Figure S8B**). In addition, each variety consisted of four replicates (plots), that is, 120 plants for each variety, and BRA900 was planted three rows in the buffer zone (**Supplementary Figure S8A**). During the identification process, 10 plants from each plot were used, and then three most seriously damaged mature leaves (leaf with ambiguous symptom or coexistence with other pests other than TSSM was not sampled; only that with typical or explicit TSSM symptom was selected) from the top, middle, and basal canopies of the plant were used for TSSM resistance identification (that was nine leaves for a single plant, approximately 90 leaves for a plot, and 360 leaves for a variety). Once the 10 plants were selected for the first time, subsequent sampling was also performed on these same plants, but the leaf sampling was random and depended on the mite damage phenotype. The mite damage scale of each sampled leaf and the number of leaves that correspond to the mite damage scale were recorded, and the final mite resistance level of each variety was calculated based on the average of 5 years of MDI analysis. Furthermore, during the identification period, no acaricide was allowed to be sprayed, while the application of fungicide (50% carbendazim wettable powder) or germicide (2% kasugamycin aqueous solution) was encouraged in case of occurrence of cassava disease. In addition, acaricide treatments were conducted in parallel with the above-mentioned mite resistance identification test, the identical varieties and plot sets were performed, the 43% bifenazate suspension concentrate was used for TSSM control as it is recognized as an effective acaricidal compound for Tetranychidae mite control in a previous study (Liang et al., 2018) and was harmless to natural enemies (Ochiai et al., 2007). The acaricide applications were calendar-based, on the 20th of each month, and sprayed 1 month after planting until 1 month before harvest, respectively (total of eight times for the whole planting season). After 10 months from planting, the yield of each variety was measured (total fresh root tube yield per plot converting to yield per hectare) in either TSSM resistance identification (acaricide-free) and acaricide application tests (yield test was performed for one time with three replicates).

### Statistical analyses

The data were analyzed using SPSS (IBM v.25). When analyzing the effects of cassava varieties on the development and reproduction of TSSM, one-way analysis of variance (ANOVA) with Tukey’s honestly significant difference multiple-comparison test was used for statistical analysis. *P<*0.05 was considered as a significant difference. All data were firstly subjected to a homogeneity test and were log- or square root-transformed if they did not meet the assumptions of normality and homoscedasticity. Moreover, a non-parametric method (Kruskal–Wallis test for independent samples) was applied when combining the three rounds of greenhouse screening results due to the utilization of categorical/qualitative data. In addition, for the field validation, a generalized linear mixed model (GLMM) was used to analyze the multiple effects such as experiment sites, acaricide application, or cassava varieties on the mite damage symptom or yield. GLMM was implemented by SPSS GENLINMIXED, with a robust estimation method for standard errors (Huber-White sandwich estimator) to account for heterogeneity of variances (Bolker et al., 2009).

Additive main effects and multiplicative interaction (AMMI) model (Gupta et al., 2021) was used to analyze the variety–environment interaction and evaluate the stability of mite resistance of each cassava variety by using Data Processing System software v. 9.50 (Tang and Zhang, 2013). Firstly, the total variation was decomposed into variety main effect (V), environment main effect (E), and variety–environment interaction effects (VEI), and then VEI values were subjected to principal component analysis, and several significant interaction principal component axes (IPCA) were obtained. Usually, IPCA1 and IPCA2 were used, where IPCA1 represents responses of variety that are proportional to the environments, which are associated with the variety × environment interaction, while IPCA2 provides information about cultivation locations that are not proportional to the environments, indicating that those are responsible of the variety × environment crossover interaction (Ajay et al., 2020). Moreover, the stability parameters (Dv and De) were available by calculating the Euclidean distance between each variety (V) or environment (E) point in the significant IPCA space and the coordinate origin. These parameters were used to evaluate the stability of the varieties in three different field test regions. The equations for Dv and De are listed below:


$$Dv=\sqrt{\sum_{k}^{\text{m}}IPCA_{vk}^{2}}$$

$$De=\sqrt{\sum_{k}^{\text{m}}IPCA_{ek}^{2}}$$

where m represents the number of significant IPCAs in the model, and IPCA_vk_ and IPCA_ek_ represent the values of vV and E on the *k*-th of IPCA, respectively.

## Results

### Large-scale identification of cassava germplasm resistant to TSSM under greenhouse condition

**Figure 2:**
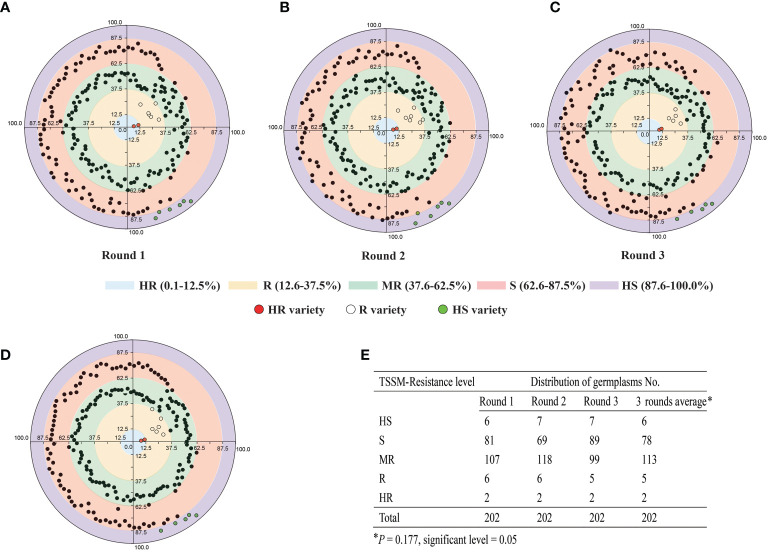
Three rounds of greenhouse identification of 202 cassava germplasm resistance to two-spotted spider mite. **(A)** First round of greenhouse identification. **(B)** Second round of greenhouse identification. **(C)** Third round of greenhouse identification. **(D)** Average of three rounds of greenhouse identifications. The different color zones indicated the different resistant levels; moreover, the values on the axes indicated the mite damage index ranges that distinguish different levels of resistance. In addition, the identified HR, R, and HS varieties were marked as red, white and green circles; other MR or S varieties were marked as black circles, respectively. **(E)** Summary data of the three rounds of greenhouse identification. The data was analyzed separately (for each round) or combined (three round average). The asterisk indicates that there is no significant difference (*P* = 0.177) of resistance levels among the three rounds of screening, and the results can be combined for analysis as validated by the non-parametric test method (Kruskal–Wallis test for independent samples).

A total of 202 cassava germplasms, including all the main cultivars in China, were subjected to three rounds of TSSM resistance identification under greenhouse condition. As shown in **Figures 2A–E** and **Supplementary Data 1**, the majority of the cassava germplasms were distributed in the S and MR regions. Nevertheless, several germplasms may shift from one resistance level to another level. More specifically, the first-round assay identified six “HS” germplasms, 81 “S” germplasms, 107 “MR” germplasms, six “R” germplasms, and two “HR” germplasms (**Figures 2A, E**); the second-round assay identified seven “HS” germplasms, 69 “S” germplasms, 118 “MR” germplasms, six resistance “R” germplasms, and two “HR” germplasms (**Figures 2B, E**); the third-round assay identified seven “HS” germplasms, 89 “S” germplasms, 99 “MR” germplasms, five “R” germplasms, and two “HR” germplasms (**Figures 2C, E**). In addition, there was no significant difference (*P* = 0.177) of resistance levels among the three rounds of screening, and the results can be combined for analysis (**Figure 2E**). To sum up, on the average, three rounds of assays identified six “HS” germplasms, 78 “S” germplasms, 113 “MR” germplasms, five “R” germplasms, and two “HR” germplasms (**Figures 2D, E**). Furthermore, C1115 and MIANDIAN are two varieties that were always identified to be “HR”, while SC5, SC9, SC15, COLUMBIA-4D, and LIMIN are five varieties that were always identified to be “R”. Moreover, KU50, BREAD, SC205, TMS60444, and BRA900 are five varieties that were always identified to be “HS”. Hence, those 12 cassava varieties, which exhibited stable resistant performance in greenhouse screening, were used to investigate their capability in affecting the reproduction and development of TSSM in the laboratory.

### The effect of identified mite-resistant and mite-susceptible cassava germplasms on the reproduction and development of TSSM

**Figure 3:**
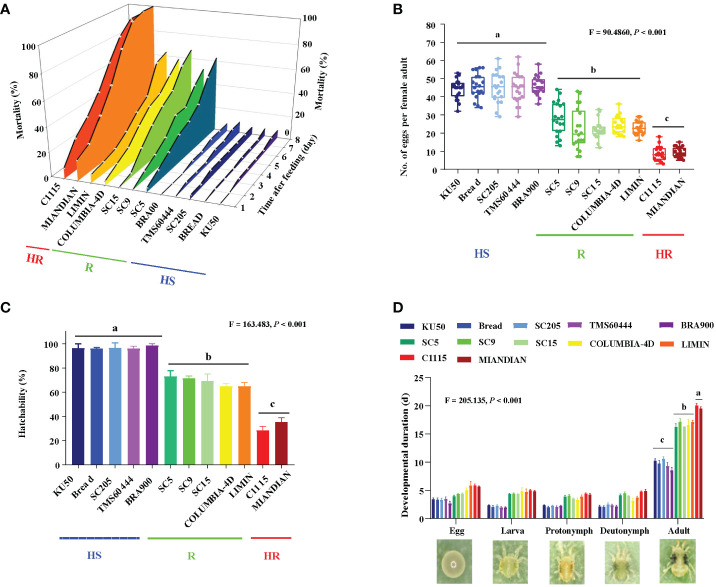
Effect on the reproduction and development of two-spotted spider mite (TSSM) while fed on different cassava varieties. **(A)** Mortalities of TSSM at 8 days post-infestation, **(B)** number of eggs per female adult, **(C)** hatchability, and **(D)** developmental duration. All data were first subjected to a homogeneity test and were log- or square root-transformed if they did not meet the assumptions of normality and homoscedasticity. Different letters indicate significant differences among batches of HR, R, and HS varieties; all analyses were based on one-way analysis of variance with Tukey’s honestly significant difference multiple comparison test (*P*< 0.05). The *F*- and *P*-values were presented in each panel.

To examine the effect of 12 cassava varieties on TSSM, the mortality, reproduction, and development of TSSM were analyzed. The results speculated that the mortality to TSSM (F_0_ generation) was significantly different among the 12 cassava varieties (**Figure 3A**). The TSSM showed very low mortalities when fed on “HS” varieties such as KU50, BREAD, SC205, TMS60444, and BRA900 (the cumulative mortality within 8 days were all below 10%). On the contrary, mite fed on “R” varieties, *i*.*e*., SC5, SC9, SC15, COLUMBIA-4D, and LIMIN, exhibited very high mortalities (the cumulative mortality within 8 days ranged from 52.45% to 67.26%). Most notably, the “HR” varieties C1115 and MIANDIAN presented the most robust lethal effect to TSSM. The mortalities of TSSM on these two varieties sharply increased after feeding and suffered 100% death within 8 dpi (**Figure 3A**). The phenomenon that cassava varieties with different resistant levels possessed significantly different capacities in inhibiting TSSM reproduction can also be seen in the aspect of fecundity (*F* = 90.486, *P*< 0.001) and hatchability (*F* = 163.483, *P*< 0.001). The average number of eggs per female adult on “HS”, “R”, and “HR” varieties were approximately 45.18, 23.86, and 9.10, respectively (**Figure 3B**). In addition, the average hatchability of TSSM on “HS”, “R”, and “HR” varieties was approximately 96.73%, 68.73%, and 31.82%, respectively (**Figure 3C**). However, the results were reversed in terms of development; both the “HR” and “R” varieties might significantly prolong the developmental duration of TSSM in each stage (*i*.*e*., egg, larva, protonymph, and deutonymph). The duration from egg to adults was 19.75 and 16.64 days, which were significantly longer compared with the HS varieties (9.81 days) (*F* = 205.135, *P*< 0.001) (**Figure 3D**). The abovementioned results suggested that resistant cassava varieties may significantly impede the normal reproduction and development of TSSM.

### Field identification of cassava varieties resistant to TSSM

**Figure 4:**
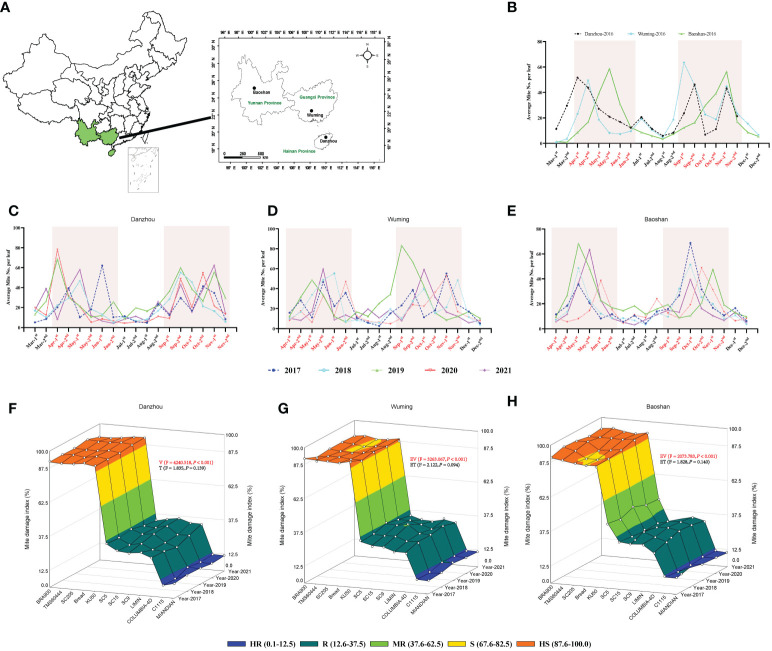
Five years of field validation of cassava varieties resistant to two-spotted spider mite (TSSM). **(A)** Geographic information of the three different sites (Danzhou, Wuming, and Baoshan) for field validation in China. **(B)** Population dynamic of TSSM at Danzhou, Wuming, and Baoshan in the year of 2016. **(C–E)** Population dynamic of TSSM at Danzhou **(C)**, Wuming **(D)**, and Baoshan **(E)** from 2017 to 2021. The shaded boxes indicate the TSSM population peak time frame of the three sites. **(F–H)** Field validation results of TSSM resistance of 12 cassava varieties at Danzhou **(F)**, Wuming **(G)**, and Baoshan **(H)**. The different color zones indicated the different resistant levels. The generalized linear mixed model was used to analyze the multiple effects such as experimental varieties and experiment time on the mite damage index, the *F*- and *P*-values were indicated within panels **(F–H)**. Furthermore, the *P*-values that represent statistical difference were marked in red (significance level = 0.05).

Cassava varieties that possessed ideal resistance to TSSM in the laboratory were used for further field identification and validation; for comparison, the “HS” varieties were also used for field test. The field experiments were carried out in three major production provinces in China (Danzhou City, Hainan Province, Wuming City, Guangxi Province, and Baoshan City, Yunnan Province) (**Figure 4A**). During the experimental period, the weather elements were recorded and analyzed. In general, the monthly average temperature, rainfall, and humidity were ranked as follows: Danzhou > Wuming > Baoshan; Danzhou and Wuming were relatively similar in weather elements (**Supplementary Figures S2–S7**), where these two sites were “wetter” than Baoshan.

Before formal field identification, a mite population survey was first conducted on “HS” cassava variety BRA900 (in the year 2016). The results showed that the mite population was correlated to the weather condition. For the years with too much rainfall (*i*.*e*., 2020 and 2021), the mite population was relatively lower than the other years and *vice versa* (**Supplementary Figures S2–S7**). In addition, although the three sites presented different dynamics of the mite population, there were two mite population peak periods during every planting season, which were from April to June and from September to November (**Figure 4B**). Furthermore, when conducting formal field identification in the following 5 years (from 2017 to 2021), the mite population peaks were also confined to those periods (**Figures 4C–E**). Based on the preliminary and formal surveys of mite population, the field identification for each variety was also conducted six times per planting season (on the 20th of April, May, June, September, October, and November).

**Figure 5:**
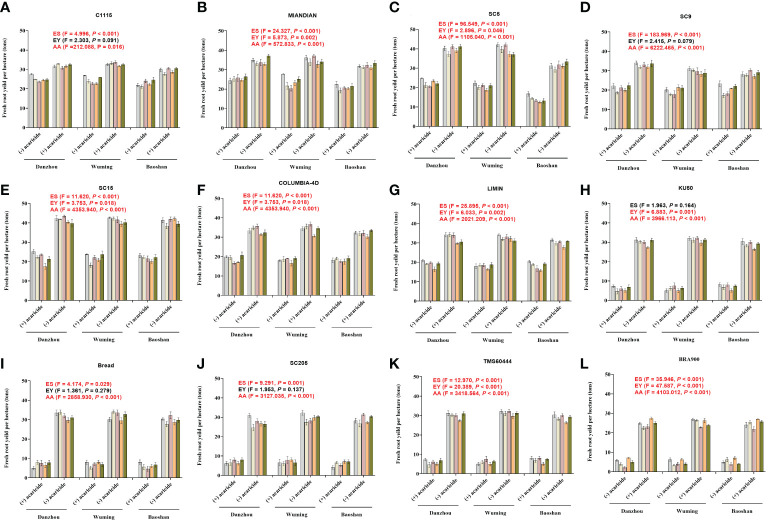
The capacity of 12 cassava varieties in reducing the yield losses during the field validation. **(A)** C1115. **(B)** MIANDIAN **(C)** SC5. **(D)** SC9. **(E)** SC15. **(F)** COLUMBIA-4D. **(G)** LIMIN. **(H)** BRA900. **(I)** SC205. **(J)** BREAD. **(K)** TMS60444. **(L)** BRA900. “+” indicates acaricide application, while “-” indicates without acaricide application. Generalized linear mixed model was used to analyze the multiple effects such as acaricide application, experiment sites, and years of experiments on the cassava yield. The *F*- and *P*-values are indicated within panels **(A–L)**. Moreover, the *P*-values that represent statistical difference are marked in red (significance level = 0.05).

The results of 5 years of field identification of a cassava variety resistant to TSSM are depicted in **Figures 4F–H** and **Supplementary Data 2**. The TSSM resistance levels of 12 cassava varieties in Danzhou was most stable, as for every tested year the identified resistance level of each variety in the field was exactly consistent with the results acquired from greenhouse analysis, namely as follows: C1115 and MIANDIAN were also identified as “HR” varieties; SC5, SC9, SC15, COLUMBIA-4D, and LIMIN were also identified as ‘R’ varieties, while KU50, BREAD, SC205, TMS60444 and BRA900 were also identified as “HS” varieties under field conditions (**Figure 4F**). The field performance in Wuming was also supposed to be “consistent” with the lab results but with minor exceptions—for example, in the years 2020 and 2021, the variety SC205 was “S”, while it was “HS” in the rest of the years (from 2017 to 2019). In addition, the resistance level of SC15 in the year 2019 was identified as “MR”, while it was “R” in other years. A similar fluctuation of TSSM resistance can also be seen in the experiment conducted in Baoshan [*i*.*e*., SC205 (2018) and BREAD (2017 and 2018)] (**Figure 4G**). However, it was noticeable that SC5 was identified to be “MR” variety for five consecutive years, while it was supposed to be “R” in the greenhouse identification (**Figure 4H**). Some cassava varieties, like C1115, MIANDIAN, COLUMBIA-4D, LIMIN, KU50, TMS60444, and BRA900, exhibited a stable and consistent resistance level across all the five years tested. In addition, the GLMM analysis for the three sites showed that a significant difference of MDI was observed between resistant and susceptible varieties, and the overall MDIs in Danzhou were significantly higher compared with those in Baoshan (**Table 2**), while the results were relatively stable across the five experiment years (**Figures 5F–H**, **Table 2**).

**Table 2:** Generalized linear mixed model (GLMM) evaluating the effect of experiment sites, cassava varieties, and experiment years on mite damage index.

Variables	Factors	Estimate	SE	t	*P*	95% CI	
						Upper	Lower
Experiment sites	Danzhou	0.940	0.3162	2.971	**0.003**	0.315	1.564
	Wuming	-0.008	0.3162	0.000	1.000	-.624	0.624
	Baoshan	0^a^					
Cassava varieties	SC5	-54.850	0.6325	-86.723	**0.000**	-56.099	-53.601
	SC9	-56.160	0.6325	-88.795	**0.000**	-57.409	-54.911
	SC15	-56.291	0.6325	-89.001	**0.000**	-57.540	-55.042
	COLUMBIA-4D	-58.566	0.6325	-92.599	**0.000**	-59.815	-57.317
	LIMIN	-58.378	0.6325	-92.302	**0.000**	-59.627	-57.130
	MIANDIAN	-78.429	0.6325	-124.004	**0.000**	-79.678	-77.180
	C1115	-78.645	0.6325	-124.345	**0.000**	-79.894	-77.396
	KU50	1.376	0.6325	2.176	**0.029**	0.127	2.625
	Bread	0.713	0.6325	1.127	0.261	-0.536	1.962
	SC205	0.330	0.6325	0.522	0.602	-0.919	1.579
	TMS60444	0.727	0.6325	1.150	0.252	-0.522	1.976
	BRA900	0^a^					
Experiment years	Year-2017	-0.101	0.3571	-.284	0.777	-0.807	0.604
	Year-2018	0.015	0.3774	0.040	0.968	-0.730	0.761
	Year-2019	1.019	0.4615	2.208	0.092	0.108	1.930
	Year-2020	0.539	0.3899	1.383	0.168	-0.231	1.309
	Year-2021	0^a^					

a This factor is redundant in GLMM analysis, so it is set to zero and as reference during pairwise comparison.

The bold values indicate the P values were lower than 0.05 and showed statistical significance.

To evaluate the capacity in reducing the losses of the yield, those 12 cassava varieties were subjected to estimation of yield under either acaricide-free or acaricide application conditions. In general, for all the varieties, it was common that the acaricide application groups all showed a significantly higher yield compared with those in the acaricide-free groups. The yields for acaricide application ranged from 23.12 to 42.36 tons/ha (**Figures 5A–L**), depending on the tested varieties, in which SC5 showed the highest yield, while TMS60444 showed the lowest yield. Conversely, without acaricide application, the yields decreased significantly; specifically, the “HR” varieties could maintain about 60%–70% of the yield (**Figures 5A, B**), and the “R” varieties could maintain about 50%–60% of the yield (**Figures 5C–G**). However, the “HS” varieties suffered the most remarkable drop in production, with approximately 70%–90% reduction of yield (**Figures 5H–L**). In addition, the GLMM analysis showed that, for each variety, the acaricide application always presented a significantly higher yield (all *P*-values lower than 0.05), while most of the experiment sites and years presented a statistical difference, depending on the TSSM resistance level (all the resistant varieties showed a significant difference in the three sites). These results indicated that the higher the TSSM resistant level, the better the performance in maintaining the yield.

**Figure 6:**
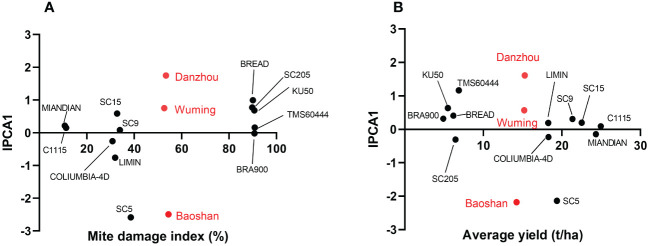
Stability analysis of the 12 cassava varieties and discrimination of regional sites by Additive main effects and multiplicative interaction biplot. **(A)** Mite damage index-based stability analysis of the 12 cassava varieties. **(B)** Yield-based stability analysis of the 12 tested cassava varieties. The 12 cassava varieties are marked with black circles, while the three regional sites are marked with red circles.

The stability and the adaptability of the 12 cassava varieties in different regions were also examined by using the AMMI model. The results speculated that variety (V), environment (E), and variety–environment interaction (VEI) would extremely significantly influence either MDI (**Supplementary Table 3**) or the cassava yield (**Supplementary Table 4**). Furthermore, in the AMMI bi-plot, the average MDI or yield was set in the X-axis, while the corresponding IPCA1s were set in the Y-axis. In the bi-plot chart, the closer the variety to the Y-axis, the more stable that the variety showed in MDI or yield and the lesser were the variation of the region sites in the TSSM resistance identification. As suggested by **Figure 6**, on one hand, when focused on MDI, the stability of the 12 cassava varieties was ranked as follows: BRA900 > SC9 > MIANDIAN > TMS60444 > C1115 > COLUMBIA-4D > SC15 > KU50 > LIMIN > SC205 > BREAD > SC5 (**Figure 6A**, **Supplementary Table S5**); on the other hand, when focused on yield, the stability of the 12 cassava varieties were ranked as follows: C1115 > MIANDIAN > COLUMBIA-4D > SC15 > LIMIN > SC9 > BRA900 > SC205 > BREAD > KU50 > TMS60444 > SC5 (**Figure 6B**, **Supplementary Table S6**). Moreover, in those two situations, Wuming was the region with the lowest variation for TSSM resistance identification, followed by Danzhou, while Baoshan showed a higher variation than the former two regions (**Supplementary Tables S7, S8**).

## Discussion

In the present study, we made considerable efforts in identifying the major cassava germplasms resistant to TSSM in China, under both greenhouse and field conditions. Finally, six cassava varieties with stable resistant levels across different planting environments were identified from 202 tested germplasms. In a previous study, four cassava varieties were identified to be resistant to a sibling mite species, the carmine spider mite, *Tetranychus cinnabarinus*, under laboratory conditions (Lu et al., 2017), but there was a lack of further field validation. Another study had also identified two cassava varieties presenting resistance to *T. cinnabarinus* under both greenhouse and field trials (Yang et al., 2020); however, the experiments were only performed in a single site and a quite limited planting season. As far as we know, this is the first attempt to conduct a large screening of cassava resistance to *Tetranychus* species. In this study, both the environmental and temporal stability were taken into account, which might largely ensure the reliability of the results.

This study provides a reliable and easy-to-handle method for screening cassava germplasm resistance to mites. In our opinion, this method is based on two factors: first, the mite damage symptom, which indicates the resistant level of cassava germplasm, must be correctly distinguished by relying on a measurable strategy rather than experience; and second, the sampled leaves should ultimately represent the actual resistance level of the overall sample, in which a quantitative method is recommended. For the first point, we have developed a computer-aided visual quantification method to determine the mite damage scale. This method can also be seen in a study regarding assessing cucumber leaf damage caused by TSSM (Uygun et al., 2020). Nevertheless, most of the studies still used the traditional way of monitoring leaf symptoms caused by pest—for example, a 1–6 damage scale was used to define the CGM resistance level by the CIAT, and 72 cultivars among the 300 cultivars consistently demonstrated lower than 3.0 of the damage ratings, indicating low to moderate CGM resistance (Bellotti et al., 2012). Hussey and Parr (1963) established a leaf damage index with 0–5 scale based on the visual evaluation of leaf infestation caused by TSSM. In another study, chlorophyll content was used to evaluate mite damage (Iatrou et al., 1995). This study offered a novel means of distinguishing the severity of TSSM damage symptoms on cassava. In addition, resistance level judgment only relies on symptoms rather than on a quantitative method, which seems empirical and not precise. Thus, parameters such as damage index or resistance index were introduced to quantitatively calculate the exact pest resistance levels of several crops like cowpea (Jackai, 1982) and potato (Fathi, 2014). Once again, for the first time, we developed a MDI-based approach that makes it more accurate and easier to evaluate cassava resistance to TSSM. With this method, stable TSSM resistance cassava varieties were excavated from 202 cassava core germplasms in China.

A total of 202 cassava germplasms, including all the main cultivars in China, were subjected to three rounds of TSSM resistance identification in 2016. On the whole, the distribution of the germplasms with different resistant levels fit the “spindle type”, as the “HR” and “HS” varieties were relatively rare, two varieties were “HR”, five varieties were “R”, and five varieties were “HS”. In comparison, most of the germplasms belonged to “MR” or “S” levels. In addition, the resistance levels of several germplasms were flexible or unstable in different rounds of identification, as one germplasm would shift from one resistance level to another level, while the 12 varieties mentioned above were steadily kept at their fixed resistant levels in any round of identification. The phenomenon of the scarcity of resistant germplasm resource is common in the insect pest resistance identification of other plant species, as cases can be found in the screening of soybean (334 genotypes) resistant to aphid (*Aphis glycines*) (Bhusal et al., 2013), pepper (50 accessions) resistant to green peach aphid (*Myzus persicae*) (Frantz et al., 2004), cotton (over 400 cultivars) resistant to whitefly (*Bemisia tabaci*) (Li et al., 2016), and maize (38 genotypes) resistant to fall armyworm (*Spodoptera frugiperda*) (Soujanya et al., 2022). In those studies, no more than seven resistant genotypes/accessions/cultivars were identified. In addition, the identification of cassava resistance to insect pest also showed this similar phenomenon—for example, field identification of about 5,500 genotypes was performed in Colombia. Approximately 75% are susceptible to whitefly (*Aleurodicus socialis*). Moreover, over 5,000 landrace cultivars in the CIAT cassava germplasm bank had been assessed, but only 6% were identified as being with low to moderate resistance to CGM. Despite the fact that considerable effort has been concentrated on screening pest-resistant cassava genotypes, few insect pest-resistant, commercial varieties are being cultivated during the past decades (Amelework and Bairu, 2022). Nevertheless, in this study, seven stably resistant varieties were identified; in particular, the good news is that although the opportunity to get highly resistant materials is usually rare during germplasm screening, we are still lucky to get two highly resistant varieties. In addition, the resistant varieties identified here are totally different from those reported in other studies, and some of them are landraces in China. Collectively, these varieties could be used as good materials for germplasm exchange or creation and will benefit future breeding programs for better management of TSSM.

The resistant cassava varieties significantly inhibited the reproduction and development of TSSM. When fed on resistant cassava varieties, the survival, oviposition, and hatchability of TSSM were all significantly inhibited, while the developmental durations were dramatically extended. Most notably, those adverse effects on TSSM were differentiated by the 12 varieties with distinct resistant levels. This phenomenon can explain the contrasting TSSM infestation phenotype of different varieties in the greenhouse as well as in the field, such that the higher the resistance level, the stronger the inhibition to TSSM. Similar results mentioned above can also be found in several pest–crop interaction studies, *i*.*e*., cotton genotype and silverleaf whitefly (Miyazaki et al., 2013), cassava varieties and papaya mealybug (*Paracoccus marginatus*) (Chen et al., 2022b) or *T. cinnabarinus* (Lu et al., 2017), rubber tree germplasms, and *Eotetranychus sexmaculatus* (Lu et al., 2016).

As a general rule, identification of crop resistance to insect pest should undergo both greenhouse and field tests; germplasms that presented excellent and consistent resistance performance can be considered as promising materials for pest control or for further breeding programs (Miyazaki et al., 2013). In the present study, the three rounds of greenhouse identification as well as the distinct effect on TSSM development and reproduction might ensure the stability and the reliability of the 12 cassava varieties to a great extent, although the resistance level of certain varieties seemed to fluctuate in certain seasons. Generally speaking, the resistance performance of the resistant or susceptible varieties in the field was quite consistent with those in the greenhouse. By contrast, fewer varieties showed a comparable performance of pest resistance in the field test compared with those in the laboratory test—for instance, there were nine aphid-resistant soybean genotypes identified in the greenhouse, but only two genotypes were identified as resistant in the field test (Bhusal et al., 2013). Zhu et al. (2018) conducted identification of resistance to *B. tabaci* using 550 cotton genotypes in greenhouse and field experiments, although the greenhouse test identified 100 resistant and susceptible genotypes, there were only 42 genotypes that showed identical resistance performance in the field test. This inconsistency may be due to the change of experimental environment—for example, the faba bean (*Vicia faba*) resistance to weed or fungi under multi-environments exhibited distinct resistance levels (Rubiales et al., 2014). In another study of *Botrytis fabae* resistance identification, field validation also revealed the instability of the resistance performance across different environments (Villegas-Fernández et al., 2009). Similar results can also be found in the identification of cassava resistance to GCM—for instance, though 300 cultivars with low to moderate resistance to CGM had been identified by the CIAT in Columbia (in the tropical lowland that possessed a prolonged dry season and endured high CGM populations) (Bellotti, 2008), only 72 cultivars were consistently demonstrated to have the same resistance level in Brazil (primarily in the northeastern semiarid regions) (Bellotti et al., 2012). This phenomenon is probably due to the environmental variability in the field, compared with the stable and normalized culture condition in the greenhouse. Abiotic stress in the field like drought, chilling, loss of applied fertilizers, and waterlogging might hinder the normal physiological development and cause the deterioration of pest resistance. However, in this study, only a minor inconsistency of resistance performance was found between greenhouse and field experiments, indicating that the resistance level of most tested varieties was stable and not inclined to be affected by environmental factors.

In this study, we state that the delicate experiment design (three rounds of greenhouse trials, five consecutive years of field validation, and three different experiment sites) ensures to get stably resistant materials. In addition, by employing the AMMI model, we also found that environment factor will significantly influence the TSSM resistance, as the variety SC5 was identified as resistant at Danzhou and Wuming but was moderately resistant at Baoshan, while the rest of the 11 varieties showed a consistent resistance performance across these three different sites. It is interesting to decipher in a future study why only SC5 exhibited an environment-dependent manner. Once again, as highlighted in the present study, most of the tested varieties showed equal resistance performance in Danzhou, Wuming, and Baoshan. As these three cities are the major areas of cassava cultivation in China, the resistant varieties identified here, to a certain extent, could probably accommodate various cassava planting environments in China; however, more regional tests are still needed to verify this hypothesis.

The yield of the 12 cassava varieties can reflect their resistance levels in the field. As acaricide was applied eight times throughout the planting season and covered all the mite population peak period of different experiment sites, we assumed that the yield we tested can represent the veritable yield of each of the tested varieties. Although C1115 and MIANDIAN were identified as “HR” varieties, they still suffered about 30% of yield losses without acaricide application. Comparatively, the “R” varieties can maintain less of the yield (50%–60%), and the TSSM caused significant yield loss to the “HS” varieties (over 80%). Interestingly, SC5 was identified as “MR” varieties in Baoshan. As expected, the yield losses were higher than those in Danzhou and Wuming, where it was identified as “R” varieties. There were rare but still some reports on insect- or mite-resistant cultivars being released to the field for pest control and to achieve a good profit. A selected number of moderate CGM-resistant cultivars had been introduced to growers by breeders and entomologists (Bellotti, 2008). Moreover, a cultivar named “Nataima-31” had been cultivated in Tolima, Colombia. This cultivar can attain a high yield of 33 t/ha (34% higher than the regional famers’ variety) without pesticide applications, and now this cultivar is being grown commercially in different regions of Colombia, Ecuador, and Brazil (CIAT, 2007). The planting area of the resistant varieties SC9, SC15, and LIMIN is expanding in China (Qin et al., 2017), which were promising main cultivars in TSSM control. Although C1115, MIANDIAN, and COLUMBIA-4D were not commercially released, they can also be considered as good material in breeding programs of mite resistance. Moreover, those varieties with distinct resistance to TSSM can promote the omics study, especially for mining the pest resistance gene, or to probe markers of pest resistance, which will, in turn, accelerate the progress of resistance breeding.

## Conclusion

In conclusion, a quantifiable identification method was used to identify cassava resistance to TSSM, and based on this method, a panel of TSSM-resistant varieties were identified under greenhouse and field conditions. This study provides promising materials for effective mite control or as good materials for deciphering the mite resistance mechanism as well as benefiting for future breeding programs of mite resistance.

## Data availability statement

The original contributions presented in the study are included in the article/**Supplementary Material**. Further inquiries can be directed to the corresponding authors.

## Author contributions

XL, QC, and YL planned and designed the research and experiments. KL, CW, MW, XY, YQ, YZ, and YG performed the laboratory experiments and analyzed the data. XL, QC, and YL wrote and edited the paper. XL and QC acquired the funds. All authors contributed to the article and approved the submitted version.
