# Supplementary material for: Identification of cassava germplasms resistant to two-spotted spider mite in China: From greenhouse large-scale screening to field validation
Source: Front Plant Sci. 2022 Dec 7;13:1054909. doi: 10.3389/fpls.2022.1054909 (PMC9768451; doi:10.3389/fpls.2022.1054909)
Supplement: Supplementary file 1 [file DataSheet_1.docx]

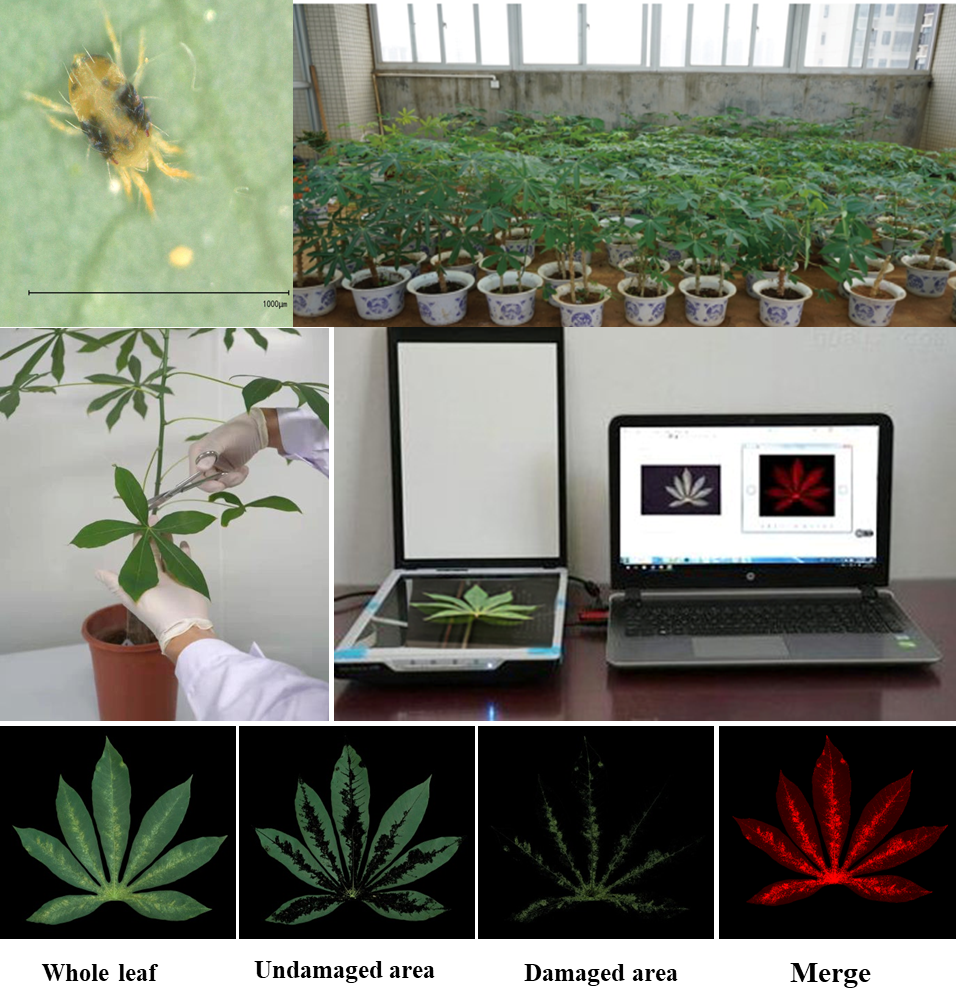


**Supplementary figure 1** The methodology of the identification of cassava germplasm resistance to TSSM. The image of the TSSM adult is presented at the top left corner. The leaf infestation rate of the cassava plant was analyzed using the Leaf Image Analyzer (YMJ-E, Daji Co. Ltd, Hangzhou, China). The four panels at the bottom indicated the original whole leaf, the visualized undamaged leaf area, the visualized damaged leaf area and merging of the damaged and undamaged areas. The damaged areas are highlighted in bright red.

**Supplementary figure 2** Elements of climate at Danzhou, Wuming and Baoshan in 2016.

**Supplementary figure 3** Elements of climate at Danzhou, Wuming and Baoshan in 2017.

**Supplementary figure 4** Elements of climate at Danzhou, Wuming and Baoshan in 2018.

**Supplementary figure 5** Elements of climate at Danzhou, Wuming and Baoshan in 2019.


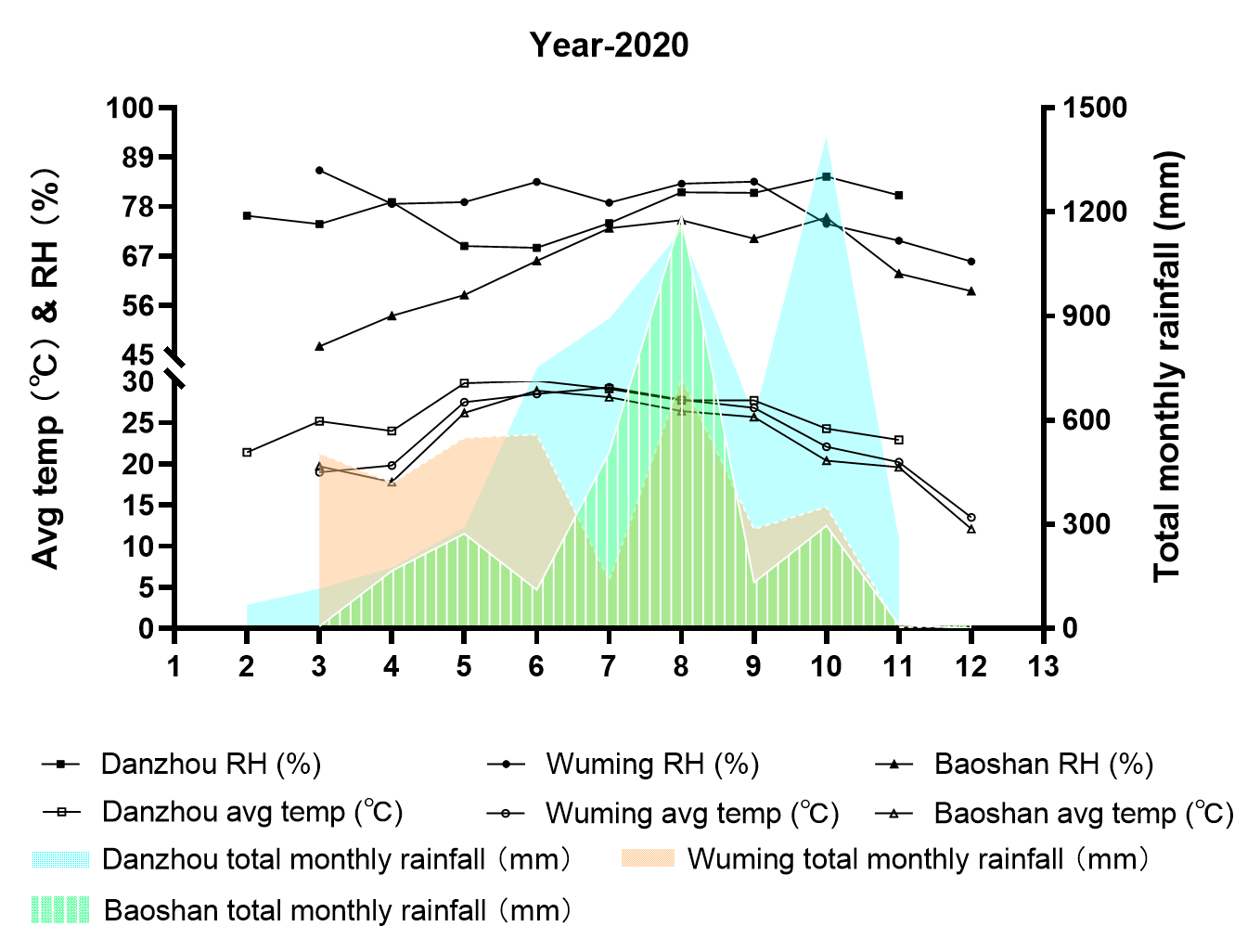


**Supplementary figure 6** Elements of climate at Danzhou, Wuming and Baoshan in 2020.


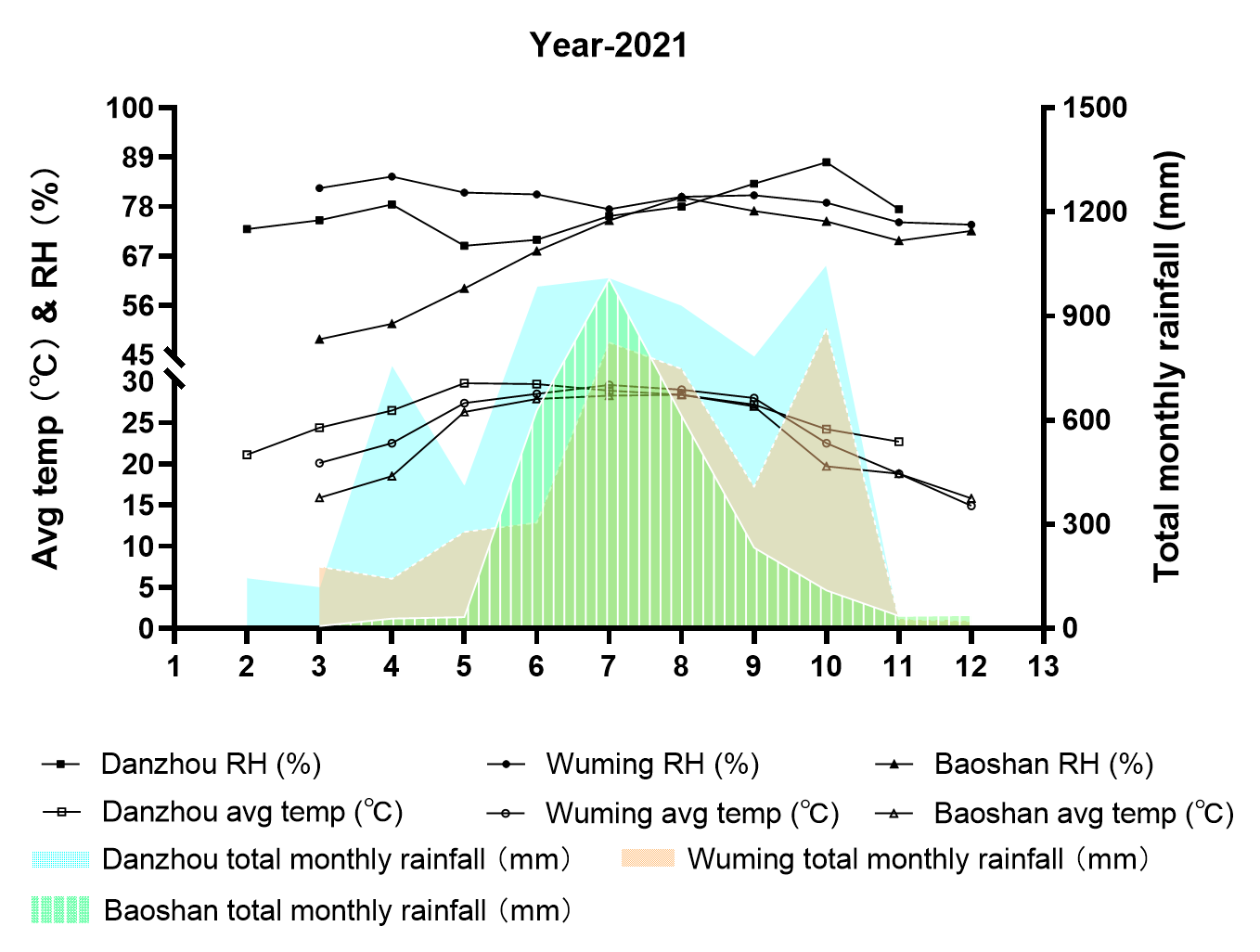


**Supplementary figure 7** Elements of climate at Danzhou, Wuming and Baoshan in 2021.


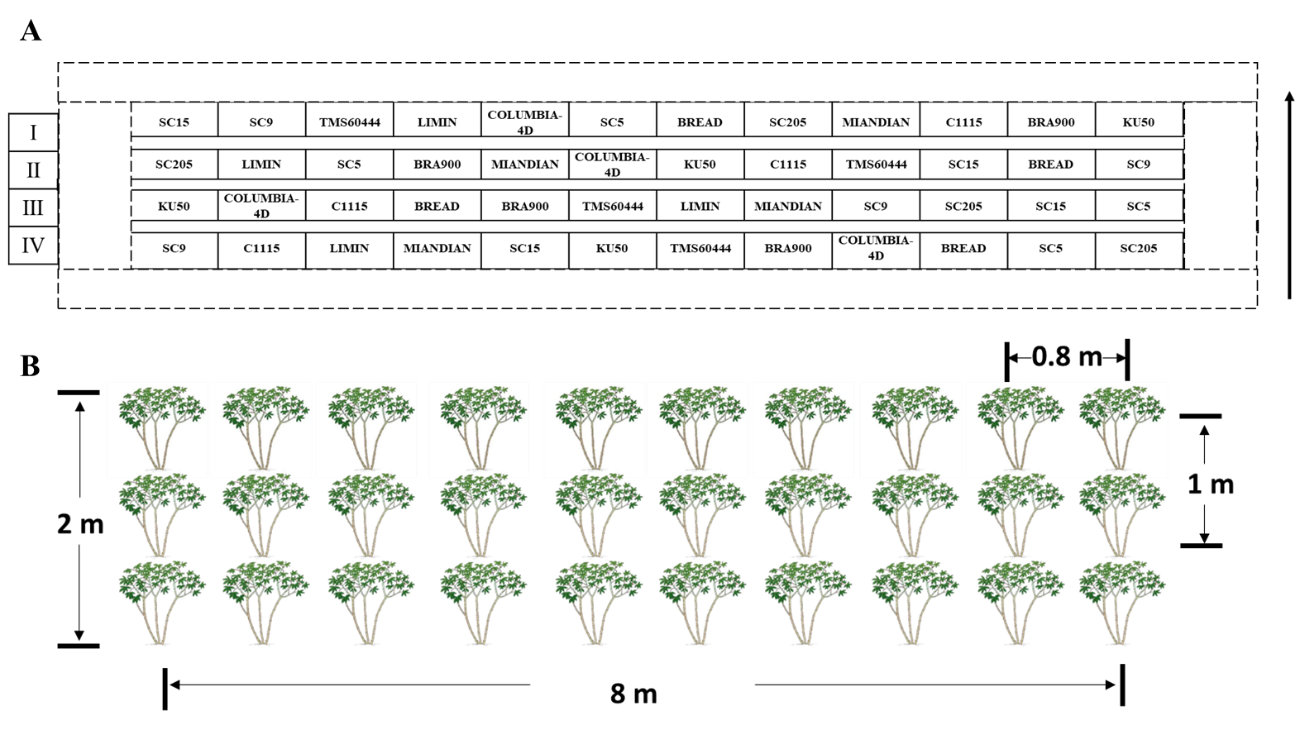


**Supplementary figure 8** (**A**)The field validation was based on randomized complete block design with 4 blocks (4 replicates for each variety). The dashed boxes indicated the buffer zones (planting with BRA900 for 3 rows), and the arrow indicated the direction of soil fertility in the field. (**B**) The area and row spacing of each plot
