# Supplementary material for: Identification of cassava germplasms resistant to two-spotted spider mite in China: From greenhouse large-scale screening to field validation
Source: Front Plant Sci. 2022 Dec 7;13:1054909. doi: 10.3389/fpls.2022.1054909 (PMC9768451; doi:10.3389/fpls.2022.1054909)
Supplement: Supplementary file 2 [file DataSheet_2.docx]

**Supplementary Table 1** Basic information of the experimental fields

| **No.** | **Location** | **Longitude and latitude** | **Soil Texture**  **Class** | **Organic Matter (%)** | **Soil pH** | **Mineral elements** | | | | |
| --- | --- | --- | --- | --- | --- | --- | --- | --- | --- | --- |
|  |  |  |  |  |  | Mg^2+^ | Ca^2+^ | K^+^ | Na^+^ | Cl- |
| 1 | Danzhou, Hainan Province | 109.508°E  19.506°N | Clay Loam | 12.8 | 6.58 | 4.23 | 5.26 | 0.78 | 7.26 | 1.96 |
| 2 | Wuming, Guangxi Province | 108.272°E  23.412°N | Clay Loam | 16.7 | 6.72 | 3.96 | 5.87 | 1.24 | 5.78 | 2.38 |
| 3 | Baoshan, Yunnan Province | 98º53′03″E,  24º57′55″N | Sandy clay Loam | 19.2 | 6.89 | 2.78 | 6.32 | 1.06 | 6.13 | 1.46 |

**Supplementary Table 2** Number and duration of field trial seasons in each regional sites

| **Location** | **Years** | **Date Initiated** | **Termination /Harvest** | **Total Duration** |
| --- | --- | --- | --- | --- |
| Danzhou | 2017 | February 20^th^ | November 23^rd^ | 10 months |
|  | 2018 | February 5^th^ | November 7^th^ | 10 months |
|  | 2019 | February 15^th^ | November 14^th^ | 10 months |
|  | 2020 | February 22^th^ | November 22^nd^ | 10 months |
|  | 2021 | February 11^th^ | November 13^th^ | 10 months |
| Wuming | 2017 | March 13^th^ | December 15^th^ | 10 months |
|  | 2018 | March 15^th^ | December 16^th^ | 10 months |
|  | 2019 | March 20^th^ | December 22^nd^ | 10 months |
|  | 2020 | March 13^th^ | December 15^th^ | 10 months |
|  | 2021 | March 20^th^ | December 23^rd^ | 10 months |
| Baoshan | 2017 | March 22^th^ | December 23^rd^ | 10 months |
|  | 2018 | March 21^th^ | December 22^nd^ | 10 months |
|  | 2019 | March 13^th^ | December 15^th^ | 10 months |
|  | 2020 | March 20^th^ | December 23^rd^ | 10 months |
|  | 2021 | March 21^th^ | December 22^nd^ | 10 months |

**Supplementary Table 3** ANOVA of mite damage index and AMMI analysis for the tested cassava varieties

| **Source of variance** | **Degree of freedom (df)** | **Sum of square (SS)** | **Mean square (MS)** | **F-value (F)** | **Probility** |
| --- | --- | --- | --- | --- | --- |
| Total | 179 | 187621.6748 | 1048.1658 | / | / |
| Treatment | 35 | 187220.5517 | 5349.1586 | 1920.3055 | 0.0001 |
| Variety (V) | 11 | 186515.5809 | 16955.9619 | 6087.0557 | 0.0001 |
| Environment (E) | 2 | 94.7301 | 47.3651 | 17.0037 | 0.0031 |
| V-E interaction | 22 | 610.2408 | 27.7382 | 9.9578 | 0.0001 |
| Error | 144 | 401.1231 | 2.7856 | / | / |

**Supplementary Table 4** ANOVA of yield and AMMI analysis for the tested varieties

| **Source of variance** | **Degree of freedom (df)** | **Sum of square (SS)** | **Mean square (MS)** | **F-value (F)** | **Probility** |
| --- | --- | --- | --- | --- | --- |
| Total | 179 | 11807.5924 | 65.9642 | / | / |
| Treatment | 35 | 11362.5082 | 324.6431 | 105.0332 | 0.0001 |
| Variety (V) | 11 | 11006.7874 | 1000.6170 | 323.7339 | 0.0001 |
| Environment (E) | 2 | 41.5751 | 20.7875 | 6.7255 | 0.0016 |
| V-E interaction | 22 | 314.1458 | 14.2794 | 4.6199 | 0.0001 |
| Error | 144 | 445.0842 | 3.0909 | / | / |

**Supplementary Table 5** Mite damage index, IPCA scores, and stability parameters of tested cassava varieties

| **Variety** | **MDI (%)** | **IPCA1** | **IPCA2** | **Stability parameter** | **Dv rank** | **MDI rank** |
| --- | --- | --- | --- | --- | --- | --- |
| BRA900 | 90.732 | -0.021 | 0.935 | 0.979 | 1 | 2 |
| Bread | 90.086 | 0.993 | 0.777 | 0.455 | 11 | 4 |
| C1115 | 10.734 | 0.213 | -0.669 | 0.792 | 5 | 12 |
| COLUMBIA-4D | 30.864 | -0.259 | -1.013 | 0.645 | 6 | 10 |
| KU50 | 90.667 | 0.684 | 0.193 | 0.509 | 8 | 3 |
| LIMIN | 31.967 | -0.764 | -0.386 | 0.494 | 9 | 9 |
| MIANDIAN | 11.315 | 0.151 | -0.287 | 0.886 | 3 | 11 |
| SC15 | 32.815 | 0.584 | -0.894 | 0.554 | 7 | 8 |
| SC205 | 89.804 | 0.772 | 0.571 | 0.491 | 10 | 5 |
| SC5 | 38.607 | -2.587 | 0.481 | 0.174 | 12 | 6 |
| SC9 | 33.998 | 0.079 | -0.311 | 0.97 | 2 | 7 |
| TMS60444 | 90.858 | 0.157 | 0.602 | 0.844 | 4 | 1 |

**Supplementary Table 6** Average yield, IPCA scores, and stability parameters of tested cassava varieties

| **Variety** | **Average yield (tons/ha)** | **IPCA1** | **IPCA2** | **Stability parameter** | **Dv rank** | **Yield rank** |
| --- | --- | --- | --- | --- | --- | --- |
| BRA900 | 4.886 | -0.325 | 0.053 | 0.41 | 9 | 12 |
| Bread | 6.001 | -0.634 | 0.352 | 0.504 | 8 | 10 |
| C1115 | 24.947 | 0.04 | -0.261 | 0.932 | 1 | 1 |
| COLUMBIA-4D | 18.145 | -0.141 | 0.056 | 0.816 | 4 | 7 |
| KU50 | 5.458 | 0.951 | -0.295 | 0.373 | 10 | 11 |
| LIMIN | 18.265 | -0.189 | -0.497 | 0.798 | 5 | 6 |
| MIANDIAN | 24.331 | 0.101 | -0.428 | 0.768 | 6 | 2 |
| SC15 | 22.501 | -0.167 | -0.042 | 0.902 | 2 | 3 |
| SC205 | 6.568 | -0.419 | 0.547 | 0.542 | 7 | 9 |
| SC5 | 19.353 | 2.142 | 0.382 | 0.282 | 12 | 5 |
| SC9 | 21.349 | -0.195 | -0.588 | 0.88 | 3 | 4 |
| TMS60444 | 6.851 | -1.163 | 0.721 | 0.361 | 11 | 8 |

**Supplementary Table 7** Mite damage index, IPCA scores, and stability parameters of the region sites

| **Region** | **MDI (%)** | **IPCA1** | **IPCA2** | **Stability parameter** | **De rank** | **Region rank** |
| --- | --- | --- | --- | --- | --- | --- |
| Danzhou | 53.4262 | 1.74517 | 1.34 | 0.891 | 2 | 2 |
| Wuming | 52.7098 | 0.74964 | -1.75 | 0.926 | 1 | 3 |
| Baoshan | 54.4763 | -2.49481 | 0.411 | 0.537 | 3 | 1 |

**Supplementary Table 8** Average yield, IPCA scores, and stability parameters of the region sites

| Region | Average yield (tons/ha) | IPCA1 | IPCA2 | Stability parameter | De rank | Region rank |
| --- | --- | --- | --- | --- | --- | --- |
| Danzhou | 15.3 | 1.61 | -0.817 | 0.914 | 2 | 1 |
| Wuming | 15.2 | 0.57 | 1.12 | 0.953 | 1 | 2 |
| Baoshan | 14.2 | -2.18 | -0.308 | 0.671 | 3 | 3 |
